# The Progression of Essential Tremors: Illustrative Videos

**DOI:** 10.5334/tohm.608

**Published:** 2021-03-24

**Authors:** Margaret McGurn, Nikki Delgado, Nora Hernandez, Elan D. Louis

**Affiliations:** 1 Department of Neurology, The University of Texas Southwestern Medical Center, Dallas, TX, USA

**Keywords:** Essential tremor, progression, clinical, phenotype

## Abstract

**Background:**

Essential tremor (ET) is a progressive neurological disease whose natural history is one of progressive increase in tremor severity over time; surprisingly though, there are no published videotape diaries that visually and tangibly portray this progression over time.

**Phenomenology:**

Progressive, stepwise increase in limb tremor severity over a ten-to-fifteen-year period in three patients with ET.

**Educational value:**

We hope that this brief visual diary will serve as a useful teaching tool for students, primary care physicians, and neurologists to “see with their own eyes” the extent of change that can occur in the ETs.

Essential tremor (ET) is a disease or a family of diseases whose natural history is one of progressive increase in tremor severity over time. As such, upper limb kinetic tremor increases in amplitude and becomes more disabling, and tremor may spread to involve other body regions, such as the cranial structures. In a cohort of 164 ET cases who were followed regularly by telephone at six to nine month intervals for up to 5.25 years, a majority reported that their condition had worsened at one-half or more of their follow-up evaluations [1]. Furthermore, one-quarter of these cases reported a worsening in tremor severity at each follow-up evaluation [1]. These patient reports of progressive worsening tremor have been validated in a study that examined tremor severity in two samples of ET patients with ≥5 years of follow-up [2]. There was an increase in total tremor score (TTS) by 0.5 or more points in 23 (95.8%) out of 24 cases in the first sample and 11 (73.3%) of 15 cases in the second sample [2]. The study estimated the average annual increase in tremor severity to be 3.1% – 5.3% and the median annual increase to be 1.8% – 2.0% [2]. Despite this pattern of progressive worsening, to our knowledge, there are no published videotape diaries that visually and tangibly demonstrate this progression of the ETs over time. Here, we provide videos of three patients with progressive, stepwise worsening upper limb tremor severity over a ten-to-fifteen-year time period (***Video 1***). At each time point, TTS (range 0–46) was assigned based on ratings (0, 0.5, 1, 1.5, 2, 3, 4) of kinetic and postural tremor during twelve motor tasks [3]. In addition to visually documenting and quantifying this progressive decline, we wish to highlight an additional feature, which is that the rate of change in TTS was not linear. To our knowledge, this nonlinearity has not been explicitly documented previously. We would like to point out that the three cases represented here are a convenience sample of three cases who had detailed longitudinal, prospective data, but that they do not represent the full spectrum of ET and do not represent the full panoply of natural histories that exist in ET. We hope that this brief visual diary will serve as a useful teaching tool for students, primary care physicians, and neurologists to “see with their own eyes” the extent of change that can occur in the ETs.

**Video 1:** Three ET Patients with Progressively Worse Tremor. *Patient 1.* This right-handed woman was 58 years old at baseline. There were five videotaped neurological examinations (T1 – T5). At T1 and T2, she was taking primidone (1,500 mg daily) and propranolol (160 mg daily). She underwent bilateral deep brain thalamic stimulation (DBS) surgery in 2015 and stopped her tremor medications at that point; the stimulator was turned off during the T3, T4, and T5 evaluations. The tremor ratings for certain movement tasks are listed in ***Table 1***. During the wing beat position, there is mild dystonic posturing of the thumb at T4 (right) and T5 (left). *Patient 2.* This right-handed woman was 66 years old at baseline. There were five videotaped neurological examinations (T1 – T5). The tremor ratings for certain movement tasks are shown in ***Table 1***. *Patient 3.* This right-handed woman was 66 years old at baseline. There were three videotaped neurological examinations (T1 – T3). At T1, T2, and T3, she was taking primidone (150 mg daily) and pindolol (10 mg daily). She had two bilateral DBS surgeries in 2007 and 2009. The tremor ratings are shown in ***Table 1***.

**Table 1:** Tremor ratings for three patients with progressively worse tremor over time.


PATIENT 1	T1	T2	T3	T4	T5

Date of exam	6/23/2005	9/9/2014	4/4/2016	10/25/2017	8/1/2019

Year of disease	33	42	44	45	47

Arm extension (R)	**1**	**0.5–1**	**1.5–2**	**2**	**3**

Wing beat (R)	– ^a^	**1**	**1.5**	**2**	**3**

Finger-nose-finger (R)	**1**	**1.5**	**2**	**3**	**3–4**

Pouring (R)	**2**	1.5	**2**	**3**	**4**

Drinking (R)	**1.5–2**	1	**3**	**3**	**4**

Drawing spirals (R)	**1.5–2**	1.5	**3**	3	**3**

TTS	17.5	19	29.5	30	34.5

**PATIENT 2**	**T1**	**T2**	**T3**	**T4**	**T5**

Date of exam	11/17/2004	1/16/2011	2/11/2015	9/1/2016	4/17/2018

Year of disease	51	58	62	63	65

Finger-nose-finger (L)	**0.5**	**1–1.5**	**2**	2	**2**

Drawing spirals (L)	**1**	2	**2**	**2–3**	3

TTS	16.5	22.5	25.5	30.5	30.5

**PATIENT 3**	**T1**	**T2**	**T3**		

Date of exam	12/20/2003	11/12/2014	6/8/2016		

Year of disease	46	57	59		

Arm extension (R)	**1**	**1.5–2**	**3**		

Finger-nose-finger (R)	**1.5**	**2–3**	**3–4**		

Pouring (R)	**2**	**3**	**4**		

Drawing spirals (R)	**1.5**	**3**	**3**		

TTS	25.5	33	45		


^a^ Video not available. R, right. L, left. TTS, total tremor score. Bolded scores indicate the task is shown in the video.

All patients were enrolled in research studies that were approved by our institutional ethics board, and all patients signed written and informed consent to be videotaped and to have their videotapes published for educational purposes.
